# Detection Rate and Variability in Measurement of Mandibular Incisive Canal on Cone-Beam Computed Tomography: A Study of 220 Dentate Hemi-Mandibles from Italy

**DOI:** 10.3390/jimaging8060161

**Published:** 2022-06-07

**Authors:** Andrea Borghesi, Diego Di Salvo, Pietro Ciolli, Teresa Falcone, Marco Ravanelli, Davide Farina, Nicola Carapella

**Affiliations:** Department of Medical and Surgical Specialties, Radiological Sciences and Public Health, University of Brescia, ASST Spedali Civili of Brescia, Piazzale Spedali Civili, 1, I-25123 Brescia, Italy; diegodisalvo@gmail.com (D.D.S.); p.ciolli@unibs.it (P.C.); t.falcone@unibs.it (T.F.); marco.ravanelli@unibs.it (M.R.); davide.farina@unibs.it (D.F.); nicola.carapella@gmail.com (N.C.)

**Keywords:** mandibular incisive canal, mandible, cone-beam computed tomography, observer variations

## Abstract

The mandibular incisive canal (MIC) is a small bony channel located in the interforaminal region; it represents the anterior continuation of the mandibular canal. Cone-beam computed tomography (CBCT) is the most commonly utilized radiological technique for assessing the MIC. The main purpose of this study was to evaluate the detectability and variability in measurements of the MIC on CBCT. A total of 220 dentate hemi-mandibles were retrospectively selected for this study. For each hemi-mandible, the detectability, diameter, and distance of the MIC from anatomical landmarks (cortical plates and tooth apices) were evaluated in consensus by two observers. The analysis was performed at four different levels (first premolar, canine, lateral incisor, and central incisor) and was repeated after one month. The variability of MIC measurements was expressed as the coefficient of repeatability (CR), obtained from the Bland–Altman analysis. The MIC detection rate reduced from the first premolar to the central incisor (from 82.3% to 0.5%). The CR of MIC measurements (diameter and distances from anatomical landmarks) was ≤0.74 mm. Although the MIC is difficult to detect in a non-negligible percentage of cases, the limited variability in measurements confirms that CBCT is an effective technique for the assessment of the MIC.

## 1. Introduction

The mandibular incisive canal (MIC) is a small bony channel located in the interforaminal region of the mandible (i.e., the anterior mandibular area between the two mental foramina) [1]. The MIC arises from the anterior loop of the mandibular canal and proceeds anteriorly within the spongiosa of the mandible towards the incisor region below the apices of the teeth [2,3,4]. 

The MIC represents the anterior continuation of the mandibular canal beyond the mental foramen and contains the intraosseous extension of the inferior alveolar neurovascular bundle, particularly the mandibular incisive nerve [5]. The mandibular incisive nerve is the terminal branch of the inferior alveolar nerve, and it provides innervation to the mandibular anterior teeth, including the first premolars [4,5,6,7]. 

The anterior mandible, specifically the interforaminal region, is generally considered a safe zone for oral and dental surgical procedures, such as bone graft harvesting and implant surgery, because of the small caliber of neurovascular structures located in this area [8]. However, in a non-negligible percentage of cases, complications can occur during or after surgical procedures due to iatrogenic injury to the neurovascular bundle located within the MIC [1,9,10,11,12]. 

The most common complications due to surgical damage to the neurovascular bundle of the MIC are discomfort, pain, and neurosensory disturbances in the anterior mandible [1,5,9,10,11]. Reported complications are usually transient, although, in some cases, they can persist for a long period [1,9,10,11]. Therefore, in order to avoid iatrogenic damage to the MIC, adequate information about the presence, size, and position of the MIC is essential during the treatment planning of surgical procedures involving the anterior mandible.

**Figure 1:**
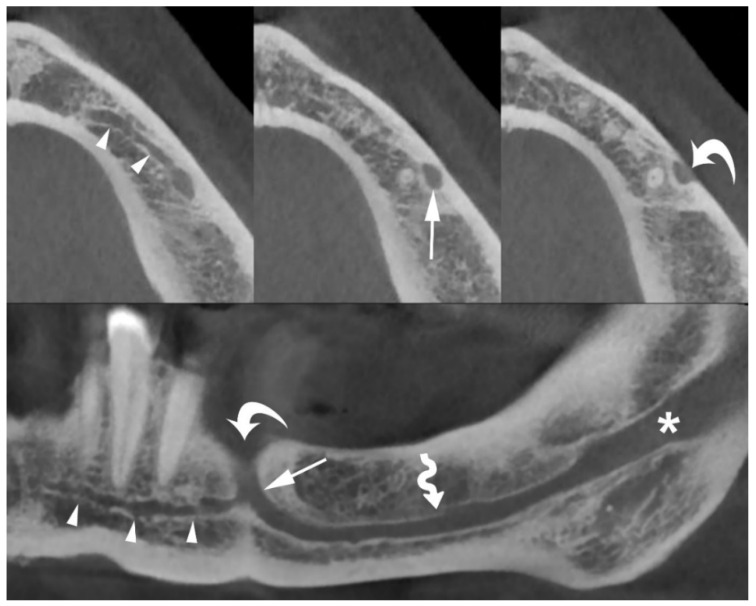
Axial (**top**) and panoramic (**bottom**) cone-beam computed tomography (CBCT) images of the left hemi-mandible demonstrate that the mandibular incisive canal (MIC) (arrowheads) originates from the anterior loop of mandibular canal (arrows) and proceeds towards the midline below the apices of the teeth. The mental foramen (curved arrows), mandibular canal (wavy arrow), and mandibular foramen (asterisk) are also shown.

In clinical practice, the most commonly used radiological technique for morphological assessment of the MIC is cone-beam computed tomography (CBCT) [3,4,5,13,14,15,16,17,18,19,20,21,22,23] (Figure 1).

Although other imaging techniques could be used in the anatomical evaluation of the mandible [24,25,26,27], the main advantages that make CBCT particularly suitable for accurate morphological analysis of the MIC are its high spatial resolution and the limited radiation dose delivered to patients [3,4,5]. 

The high spatial resolution of CBCT provides detailed anatomical information regarding the canals and foramina located within the mandible, and has revealed many anatomical variants [28,29]. The frequency of these anatomical variations in CBCT scans varies among ethnic groups, with higher incidence among Asians than Caucasians [28]. However, unexpected anatomical variants of foramina and canals of the mandible have also been described in Caucasians [28,29]. Therefore, preoperative planning based on CBCT should help oral and dental surgeons avoid iatrogenic complications during any surgical procedures involving the mandible.

Several studies have investigated the presence, size, and position of the MIC on CBCT images [1,2,3,4,5,13,14,15,16,17,18,19,20,21,22,23]. However, to our knowledge, no CBCT study has evaluated the presence, size, and distance of the MIC from adjacent anatomical landmarks, and the observer variability of MIC measurements, in the dentate mandibles of Caucasian (Italian) patients.

Therefore, the main purpose of this CBCT study was to retrospectively evaluate the detectability, diameter, and distance of the MIC from anatomical landmarks at four different reference levels (first premolar, canine, lateral incisor, and central incisor), and the observer variability of MIC measurements (i.e., diameter and distance of the MIC from adjacent anatomical landmarks). Additionally, we evaluated sex-related differences in the detectability and diameter of the MIC.

## 2. Materials and Methods

### 2.1. Patient and Hemi-Mandible Selection

The study sample included mandibular CBCT scans of patients referred to our radiology department between January 2012 and May 2013. The sample size was selected based on the following criteria: (a) Caucasian (Italian) patients, (b) patients aged 18 years or older, (c) dentate patients (at least in the anterior mandible, from the right first premolar to the left first premolar), and (d) no evidence of motion or metal artifacts on CBCT images.

Patients with pathological disorders, such as trauma, cysts, tumors, condensing osteitis, osteomyelitis, and osteonecrosis, or a history of surgical procedures, such as bone graft harvesting and implant placement, in the anterior mandible were excluded.

This study was notified to our local ethics committee as a retrospective analysis. Given the retrospective nature of this analysis, the need for informed consent was waived.

### 2.2. Image Acquisition

All CBCT scans were obtained using a dedicated CBCT scanner (NewTom 5G^®^, QR, Verona, Italy) at a tube voltage of 110 kVp; the exposure time and tube current varied depending on the selected field of vision (15 × 5 or 15 × 12 cm). 

**Figure 2:**
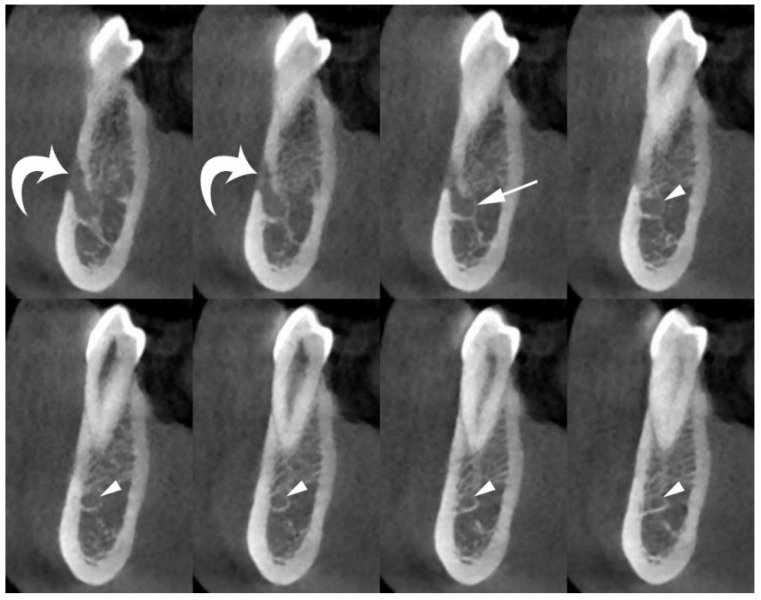
Cross-sectional CBCT images show the origin and position of the MIC (arrowheads) within the spongiosa of the mandible, and its relationships with adjacent anatomical landmarks (cortical plates and first premolar apex). The mental foramen (curved arrows), and anterior loop of mandibular canal (arrow) are also shown.

The acquired volume was reconstructed as axial images with a voxel size ≤ 200 µm. Using a dedicated tool for dental planning, this high-resolution dataset was further reconstructed as multiplanar images (200-µm-thick), perpendicular to the curvature of the mandible (cross-sectional images). Cross-sectional images are the most appropriate reconstructions to evaluate the presence and size of the MIC and its relationships with the adjacent mandibular anatomical landmarks (Figure 2).

Additionally, multiplanar reconstructions parallel to the curvature of the mandible (panoramic images) with thicknesses of 0.5 and 20 mm were performed.

### 2.3. Image Analysis

The CBCT images were analyzed using the department’s picture archiving and communication system (Intellispace PACS Radiology, Philips, Amsterdam, The Netherlands). The parameters analyzed on the hemi-mandibles selected for this retrospective study were as follows: (a) detection rate of the MIC; (b) the maximum diameter of the MIC; (c) the vertical distance between the superior margin of the MIC and the tooth apex; (d) the vertical distance between the inferior margin of the MIC and the inferior cortical plate; (e) the horizontal distance between the buccal margin of the MIC and the buccal cortical plate; and (f) the horizontal distance between the lingual margin of the MIC and the lingual cortical plate. 

We selected these parameters as they are of considerable importance to define a safe zone for surgical procedures involving the anterior mandible, such as bone graft harvesting and implant surgery.

**Figure 3:**
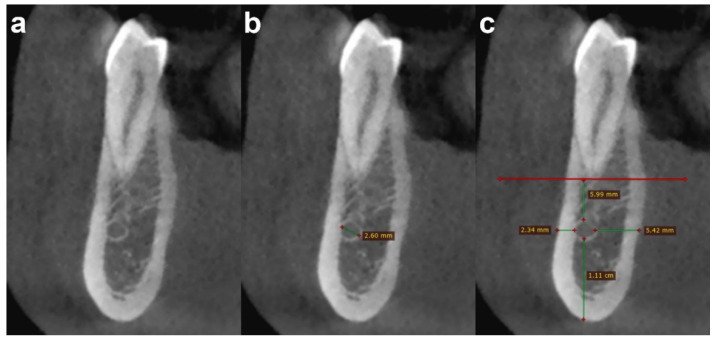
Cross-sectional CBCT images show an example of the steps of image analysis of the MIC at the first premolar level, as follows: (**a**) detection; (**b**) diameter measurement; and (**c**) distance measurement from adjacent anatomical landmarks (cortical plates and first premolar apex).

All MIC parameters were evaluated on cross-sectional images at four anatomical reference levels (first premolar, canine, lateral incisor, and central incisor) by two observers (A.B. and D.D.S., with 10 and 2 years of experience in dental CBCT, respectively) (Figure 3). The two observers performed the image analysis in a consensus reading (i.e., performed together, not independently). We used this method of analysis to simulate a surgical planning situation (i.e., a preoperative imaging assessment by a surgical team).

Additionally, to assess the variability of MIC measurements (i.e., diameter and distances from the cortical plates and teeth apices), the same observers repeated the analysis after one month. 

### 2.4. Statistical Analysis

Data are presented as number (%) or median and interquartile range (IQR) for non-normally distributed data, and as mean ± standard deviation for normally distributed data.

To define the minimum required number of hemi-mandibles, sample size calculation was performed using the method proposed by Lu et al. [30]. With a statistical power of 80%, a type I error of 0.05, and predefined values of mean difference (0.10 mm), standard deviation of differences (0.60 mm), and maximum acceptable difference between measuremments (1.50 mm), we found that the minimum required number of hemi-mandibles was 169. We set a maximum acceptable difference between measurements of 1.50 mm to ensure an adequate safety margin.

The chi-square test and the Mann–Whitney *U* test were utilized to analyze sex differences in the detectability and diameter of the MIC at the four anatomical reference levels. The findings in the first round of evaluation were considered for this analysis.

The Bland–Altman method was utilized to calculate the observer variability of MIC measurements (i.e., diameter and distances from cortical plates and teeth apices) at the four predefined anatomical reference levels. For each parameter, the differences in measurements were expressed as the absolute difference between each pair of measurements, divided by the mean of the two measurements. From the Bland–Altman analysis, we obtained the mean difference and coefficient of repeatability (CR) [31]. The mean difference, also called the “mean”, is the bias, and it represents the systematic error related to a measurement (i.e., the mean positive or negative variation of the second measurement compared to the first measurement). The CR was calculated as 1.96 times the standard deviation of the differences between the measurements and is a measure of the 95% limits of agreement. The CR provides a value, below which the difference between two repeated measurements should fall with a probability of 95%. Therefore, the CR represented the maximum expected difference between two repeated measurements.

Statistical analysis was performed using MedCalc^®^ Statistical Software (MedCalc Software Ltd., version 20.104, Ostend, Belgium; https://www.medcalc.org, accessed on 3 June 2022).

## 3. Results

Based on the study selection criteria, 110 Caucasian (Italian) patients (35 men and 75 women) with 220 dentate hemi-mandibles were enrolled in this retrospective analysis. The median age of the selected patients was 44.5 years (IQR, 33–59 years). There was no significant difference in age between men (median, 41 years; IQR, 29–58 years) and women (median, 45 years; IQR, 38–59 years) (*p* = 0.200).

The MIC detection rate ranged from 82.3% (at the first premolar level) to 0.5% (at the central incisor level). Table 1 shows the detection rate of MIC at the four anatomical reference levels. Further, Table 1 shows no significant sex differences in the detection rate of MIC (*p* ≥ 0.327).

The median diameter of the MIC ranged from 1.63 mm (at the first premolar level) to 1.00 mm (at the lateral incisor level). In the only hemi-mandible where the MIC was visible at the level of the central incisor, the diameter was 1.22 mm. Table 2 shows the diameter of the MIC at the four anatomical reference levels. Further, Table 2 shows no significant sex differences in the diameter of the MIC (*p* ≥ 0.213).

Regarding the variability in measurement of MIC diameter, the mean difference ranged from 0.05 mm (at the first premolar level) to 0.03 mm (at the canine and lateral incisor levels), and the CR ranged from 0.27 mm (at the first premolar level) to 0.21 mm (at the lateral incisor level). Table 3 shows the values obtained in the two rounds of measurements, mean difference, and CR for MIC diameters obtained from the Bland–Altman analysis.

Regarding the variability in measurement of the vertical distance between the MIC and tooth apex, the mean difference ranged from −0.02 mm (at the first premolar level) to −0.04 mm (at the canine and lateral incisor levels), and the CR ranged from 0.74 mm (at the first premolar level) to 0.63 mm (at the canine level). Table 4 shows the values obtained in the two rounds of measurements, mean difference, and CR for the distance between the MIC and the tooth apex obtained from the Bland–Altman analysis.

Regarding the variability in measurement of the vertical distance between the MIC and inferior cortical plate of the mandible, the mean difference ranged from 0.02 mm (at the first premolar and canine levels) to 0.01 mm (at the lateral incisor level), and the CR ranged from 0.60 mm (at the first premolar level) to 0.45 mm (at the canine level). Table 5 shows the values obtained in the two rounds of measurements, mean difference, and CR for the distance between the MIC and the inferior cortical plate obtained from the Bland–Altman analysis.

Regarding the variability in measurement of the horizontal distance between the MIC and buccal cortical plate of the mandible, the mean difference ranged from 0.04 mm (at the lateral incisor level) to 0.02 mm (at the first premolar level), and the CR ranged from 0.65 mm (at the first premolar level) to 0.45 mm (at the canine level). Table 6 shows the values obtained in the two rounds of measurements, mean difference, and CR for the distance between the MIC and the buccal cortical plate obtained from the Bland–Altman analysis.

Regarding the variability in measurement of the horizontal distance between the MIC and lingual cortical plate of the mandible, the mean difference ranged from 0.05 mm (at the lateral incisor level) to 0.01 mm (at the first premolar level), and the CR ranged from 0.67 mm (at first premolar level) to 0.48 mm (at the lateral incisor level). Table 7 shows the values obtained in the two rounds of measurements, mean difference, and CR for the distance between the MIC and the lingual cortical plate obtained from the Bland–Altman analysis.

## 4. Discussion

In this study, we retrospectively analyzed the detectability, diameter, and distances of the MIC from anatomical landmarks (cortical plates and teeth apices), and the observer variability of MIC measurements on CBCT images. 

Although the presence, size, position, and course of the MIC on CBCT images have been previously studied by several authors, very few CBCT studies have investigated observer variability in MIC measurements [1,14,23]. To our knowledge, there are no published studies evaluating the variability of MIC measurements in Caucasians.

In our study sample, consisting of 220 dentate hemi-mandibles from 110 Caucasian (Italian) patients, the MIC detection rate progressively reduced from the first premolar to the central incisor because the MIC diameter gradually decreased from the distal to the mesial part of the anterior mandible. This finding is similar to findings in several CBCT studies [3,10,14,15,17,22].

The MIC detection rate at the first premolar level was 82.3%. Previous studies have reported that the prevalence of the MIC on CBCT images at its origin or at the first premolar root ranges from 100% to 43.9% [1,2,3,4,13,14,15,16,17,18,19,20,21,22,23]. Factors that seem to influence the detectability of the MIC are sex, age, dental status, and ethnicity [3,4,8,14,16,19,20]. With regard to the influence of sex, some authors have reported that the prevalence of the MIC on CBCT images is higher in women than in men [3,16,20]. In another CBCT study, Zhang et al. found that men and dentate patients had a higher prevalence of MIC than women and edentulous patients, respectively. However, in the present study, we did not observe any sex-related differences in the detection rate of MIC. This finding is similar to findings in other CBCT studies, which found no sex influence on the prevalence of MIC [10,22,23].

Previous studies have reported that the MIC commonly ends at the level of the canine or lateral incisor [14,19]. Zhang et al. also reported that, in dentate mandibles, only 2.2% of MICs ended at the level of the central incisors [19]. Similarly, in our study, the MIC was detected at the central incisor level in only one hemi-mandible (0.5%). In contrast, in the study by Kabak et al., the MIC reached the central incisor in 21% of cases [15].

The diameter of the MIC progressively decreases from its origin to its terminal portion [3,10,14,15,17,22]. In this study, the median diameter of the MIC at the first premolar level was 1.63 mm. Previous studies have reported that the mean diameter of MIC on CBCT images at its origin or at the first premolar level ranges from 1.47 mm to 2.80 mm [4,10,13,14,15,17,19,22]. Some CBCT studies have analyzed the influence of sex, age, and dental status on the MIC diameter [4,13,14,15,17,19,22]. Yang et al. and Zang et al. found that the MIC diameter at its origin was significantly larger in men than in women [14,19]. Gills et al. also demonstrated that older (>60 years) and edentulous patients had a larger MIC diameter compared to other groups [17]. In contrast, Zhang et al. found that the MIC diameter was larger in dentate than in edentulous patients [19]. In our study, we did not observe sex-related differences in MIC diameter. This finding is similar to findings observed in other CBCT studies [4,13,15,22].

Regarding the distances between the MIC and adjacent anatomical landmarks, such as the inferior, buccal, and lingual cortical plates and teeth apices, we found that the MIC exhibited a downward slope from the first premolar to the incisor region (the distance to the tooth apex increased and the distance to the inferior cortical plate decreased) with progressive movement away from the buccal cortical plate and towards the lingual mandibular cortical plate. This finding is similar to findings observed in several CBCT studies [1,14,17,19,22,23]. In addition, some studies reported sex-related differences in the distances of the MIC from the teeth apices and mandibular cortical plates (distances were greater in men than in women) [1,19,22,23].

Data on the observer variability of MIC measurements on CBCT images are limited, and few studies have analyzed this aspect [1,23]. The assessment of observer variability is an important aspect for any research that wants to evaluate the reliability and validity of a measurement method. In this regard, the assessment of observer variability of MIC measurements on CBCT images has relevant clinical implications, as it may help surgeons to choose the safest surgical procedure to avoid neurovascular complications.

Al-Ani et al. evaluated the intra-observer variability in distances from the MIC to the mandibular cortical plates (inferior, buccal, and lingual) on CBCT images [1]. In their population, consisting of 120 hemi-mandibles from 60 Asian patients, the measurement error of distances of the MIC from anatomical landmarks was ≤0.05 mm [1]. Yang et al. and Alshamrani et al. evaluated the inter-observer agreement of MIC measurements [14,23]. However, these authors measured the inter-observer agreement using Cohen’s kappa and the intraclass correlation coefficient [14,23]; therefore, only a coefficient of agreement was provided in their CBCT studies.

In the present study, we found, for the first time, that the mean difference (i.e., the bias) and the CR (i.e., the maximum expected difference between two repeated measurements) obtained from the Bland–Altman analysis for MIC measurement (diameter and distance from anatomical landmarks) were ≤0.05 mm and ≤0.74 mm, respectively. 

The limited variability of MIC measurements observed in our study further confirms that CBCT is an effective radiological imaging technique for the anatomical assessment of the MIC. Additionally, considering the widespread availability of CBCT scanners and the increased number of surgical procedures in the anterior mandible, our results have important clinical implications because they provide a precise topographic map of the MIC, helping dental surgeons reduce both permanent and transient iatrogenic neurosensory disturbances in the interforaminal region [1,5,9,10,11]. 

In this regard, the present study also suggests that the risk of iatrogenic damage to the MIC progressively reduces from the distal to the mesial part of the mandible as the detectability and the diameter of the MIC gradually decrease. Based on our results, we can assume that the potential risk for neurovascular complications during and after surgical procedures performed on the inferior incisor region is very low, specifically at the level of the central incisor. On the other hand, the potential risk of iatrogenic injuries to the MIC during and after surgical procedures performed on the canine and premolar regions is not negligible, specifically at the level of the first premolar, as the detectability and the diameter of the MIC may be enough to determine the onset of significant neurovascular disturbances. 

Finally, the position of the MIC progressively changes from the first premolar to the central incisor. Therefore, the intraosseous course of the MIC should be carefully evaluated during the preoperative surgical planning.

Our study had some limitations. First, this was a retrospectively study. Second, it lacked a gold standard to verify the reliability of CBCT measurements; however, previous studies have demonstrated that CBCT measurements exhibit excellent reliability compared with measurements obtained from cadaveric dissection [32,33]. Third, MIC analysis was performed by two observers in consensus; however, we opted for this method to reproduce a surgical planning situation.

In conclusion, although the MIC is difficult to detect in a non-negligible percentage of cases, the limited variability in measurements observed in our study confirms that CBCT is an effective imaging technique for the anatomical assessment of the MIC. Therefore, dental surgeons should always use CBCT whenever there is a risk of iatrogenic injury to the neurovascular bundle of the MIC during surgical procedures in the interforaminal region. 

## Figures and Tables

**Table 1:** The MIC detection rate at the four anatomical reference levels.

Site	MIC Detection Rate (%)			*p* Value *
	Man	Woman	Total	
First premolar	55/70 (78.6)	126/150 (84.0)	181/220 (82.3)	0.327
Canine	43/70 (61.4)	83/150 (55.3)	126/220 (57.3)	0.396
Lateral incisor	11/70 (15.7)	28/150 (18.7)	39/220 (17.7)	0.594
Central incisor	0/70 (0.0)	1/150 (0.7)	1/220 (0.5)	NA

Data are presented as numbers (%); * *p* values obtained by means the chi-square test; NA, not applicable.

**Table 2:** The MIC diameter at the four anatomical reference levels.

Site	MIC Diameter (mm)			*p* Value *
	Man	Woman	Total	
First premolar	1.68 (1.33–1.97)	1.54 (1.29–1.84)	1.63 (1.33–1.92)	0.213
Canine	1.22 (1.00–1.34)	1.23 (1.01–1.40)	1.22 (1.01–1.40)	0.425
Lateral incisor	1.00 (0.96–1.22)	0.92 (0.83–1.26)	1.00 (0.83–1.22)	0.387
Central incisor	-	1.22	1.22	NA

Data are presented as medians (interquartile range); * *p* values obtained by means the Mann-Whitney *U* test; NA, not applicable.

**Table 3:** Variability in measurement of MIC diameter.

Site	Round of Measurement (mm)		Mean (mm)	CR (mm)
	First	Second		
First premolar	1.63 (1.33–1.92)	1.54 (1.31–1.84)	0.05	0.27
Canine	1.22 (1.01–1.40)	1.20 (1.00–1.38)	0.03	0.24
Lateral incisor	1.00 (0.83–1.22)	1.00 (0.80–1.20)	0.03	0.21
Central incisor	1.22	1.25	NA	NA

Data are presented as medians (interquartile range) or absolute value; Mean, mean difference; CR, coefficient of repeatability; NA, not applicable.

**Table 4:** Variability in measurement of distance between the MIC and the tooth apex.

Site	Round of Measurement (mm)		Mean (mm)	CR (mm)
	First	Second		
First premolar	5.85 ± 2.58	5.89 ± 2.55	−0.02	0.74
Canine	6.58 ± 3.00	6.62 ± 3.01	−0.04	0.63
Lateral incisor	8.02 ± 3.41	8.06 ± 3.39	−0.04	0.73
Central incisor	11.95	11.84	NA	NA

Data are presented as mean ± standard deviation or absolute value; Mean, mean difference; CR, coefficient of repeatability; NA, not applicable.

**Table 5:** Variability in measurement of distance between the MIC and the inferior cortical plate.

Site	Round of Measurement (mm)		Mean (mm)	CR (mm)
	First	Second		
First premolar	8.68 ± 1.62	8.67 ± 1.62	0.02	0.60
Canine	6.79 ± 1.68	6.77 ± 1.69	0.02	0.45
Lateral incisor	6.67 ± 1.53	6.65 ± 1.53	0.01	0.48
Central incisor	6.32	6.38	NA	NA

Data are presented as mean ± standard deviation or absolute value; Mean, mean difference; CR, coefficient of repeatability; NA, not applicable.

**Table 6:** Variability in measurement of distance between the MIC and the buccal cortical plate.

Site	Round of Measurement (mm)		Mean (mm)	CR (mm)
	First	Second		
First premolar	3.12 ± 1.21	3.11 ± 1.23	0.02	0.65
Canine	4.90 ± 1.63	4.87 ± 1.64	0.03	0.45
Lateral incisor	5.23 ± 1.44	5.19 ± 1.47	0.04	0.52
Central incisor	4.06	4.02	NA	NA

Data are presented as mean ± standard deviation or absolute value; Mean, mean difference; CR, coefficient of repeatability; NA, not applicable.

**Table 7:** Variability in measurement of distance between the MIC and the lingual cortical plate.

Site	Round of Measurement (mm)		Mean (mm)	CR (mm)
	First	Second		
First premolar	5.30 ± 1.72	5.29 ± 1.71	0.01	0.67
Canine	4.72 ± 1.86	4.68 ± 1.84	0.04	0.51
Lateral incisor	5.07 ± 1.60	5.02 ± 1.63	0.05	0.48
Central incisor	5.61	5.67	NA	NA

Data are presented as mean ± standard deviation or absolute value; Mean, mean difference; CR, coefficient of repeatability; NA, not applicable.

## Data Availability

The data used to support the findings of this study are available from the corresponding author upon request.
